# Validation of an Eye–Foot Coordination Assessment Tool for Children in Dual-Task Condition

**DOI:** 10.3390/jcm14010172

**Published:** 2024-12-31

**Authors:** Karina Elizabeth Andrade-Lara, Víctor Serrano Huete, Eva Atero Mata, Juan Antonio Párraga Montilla, Julio Herrador Sánchez, Asensio Moreno Marín, Melchor Martínez Redondo, Daniel Manjón Pozas, Jesús Salas Sánchez, Manuel Lucena Zurita, José Carlos Cabrera Linares, Pedro Ángel Latorre Román

**Affiliations:** 1 Department of Musical, Plastic and Corporal Expression, University of Jaén, 23071 Jaén, Spain; karinandrade9011@gmail.com (K.E.A.-L.); evamaria.atero@ui1.es (E.A.M.); jparraga@ujaen.es (J.A.P.M.); amm00093@red.ujaen.es (A.M.M.); platorre@ujaen.es (P.Á.L.R.); 2 Department of Physical Education Didactics and Health, International University of La Rioja, 26006 Logroño, Spain; victor.serranohuete@unir.net (V.S.H.); jesus.salassanchez@unir.net (J.S.S.); 3 Department of Sports and Computer Sciences, University Pablo de Olavide, 41013 Sevilla, Spain; jahersan1@upo.es; 4 CEIP Doctor Fleming, 23500 Jódar, Spain; melchor_mr@hotmail.com; 5 Escuelas Profesionales de la Sagrada Familia, 23400 Úbeda, Spain; dmanjon@fundacionsafa.es (D.M.P.); mlucena@fundacionsafa.es (M.L.Z.); 6 Facultad de Educación, Universidad Autónoma de Chile, Temuco 1090, Chile

**Keywords:** dual paradigm, motor competence, coordination test, interference, motor task

## Abstract

**Background/Objectives**: Eye–foot coordination is essential in sports and daily life, enabling the synchronization of vision and movement for tasks like ball control or crossing obstacles. This study aimed to examine both the validity and reliability of an innovative eye–foot coordination (EFC) test in a dual-task paradigm in children aged 6–11 years and the capacity of this test to discriminate between sex and age. **Methods**: A total of 440 schoolchildren aged 6–11 years participated in this cross-sectional study. A ball control test, involving kicking and catching, was used to assess EFC. The assessment included three conditions: without interference (WI), with auditory interference (AI), and with visual interference (VI). **Results**: The ICCs per the EFC test scores were 0.975 for foot successes (95% CI = 0.961–0.983; *p* < 0.001) and 0.747 for foot mistakes (95% CI = 0.611–0.835; *p* < 0.001). The SEM for the standing successes was 3.082 (10.81%), and the MDC was 4.860 (17.05%). For the standing mistakes, the SEM was 1.551 (19.33%) and the MDC was 3.452 (43.04%). Moreover, boys had a significantly higher number of successes in the WI, AI, and VI conditions (*p* < 0.001, respectively) than girls, although girls had more mistakes than boys only in the VI condition (*p* = 0.025). **Conclusions**: The EFC test showed adequate reliability and validity. Also, the EFC test showed that performance worsened with interference regardless of sex and age, especially in girls in the VI condition.

## 1. Introduction

The mastery of fundamental motor skills is the cornerstone of motor development, providing the building blocks for participation and engagement in physical activity and sports [1]. These skills are divided into three main domains: locomotor skills (e.g., jumping, running, crawling, walking), object control skills with a focus on manipulating objects (e.g., throwing, catching, dribbling, kicking, striking), and stability skills (e.g., balance and control) [2]. Therefore, these skills form the foundation of motor competence, reflecting an individual’s proficiency in performing a variety of motor tasks that require precise control and effective coordination of the body [3].

Specifically, motor competence is defined as the ability to perform fundamental and specific motor skills in an efficient, coordinated, and controlled manner [4]. Motor competence reflects a person’s level of performance in specific skills or motor acts, involving both fine and gross motor coordination [5]. Furthermore, it also encompasses the motor control, coordination, and sensorimotor processes necessary for coordinated movement and effective physical activity [6]. In this line, this concept is crucial, as an adequate level of motor competence facilitates children’s development during daily activities and encourages the practice of physical activity [7]. Likewise, in sports, it has a direct impact on performance, as skills such as speed, agility, and coordination are essential for success [8]. In this context, Stodden’s [9] model suggested that greater motor competence in the early stages of life is associated with greater participation in physical activity. This early development has a direct relationship with the typical development of children’s motor competence and nervous system, and their participation in different physical activities in later life [10]. However, scientific evidence has shown that the levels of fundamental motor skills in children aged 3 to 10 years are below the established average, with a greater mastery of locomotor skills than of object control skills [10]. Therefore, motor competence deficiency can undermine children’s self-confidence [11] and condition their adherence to physical activity from the earliest stages [2]. In this context, given that the regular practice of physical activity has positive impacts on cognition [12] and enhances children’s motor development [13], physical education is the key piece in this connection [14]. In this regard, physical education through physical exercise and physical activity integrating diverse and structured motor experiences enhance cognitive and motor development in children [15]. This interaction has a significant impact on increasing cerebral blood flow, favouring neuroplasticity and promoting the release of neurotrophic factors, promoting the development and maintenance of cognitive skills [16]. In addition, physical education contributes to learning and the acquisition of skills that benefit the holistic development of students, such as creativity [17], executive function [18], and motor development [13].

In this line, coordination refers to the integration of sensory information (e.g., sight, touch, proprioception) with motor information to execute precise and controlled actions [19]. Boys often show greater mastery of object control skills than girls [10]. This difference underscores the role of coordination in physical education, which is often used as an indicator of motor development and as a factor in the diagnosis and treatment of motor impairments [20]. Furthermore, developing motor coordination in physical education supports integral growth and fosters motor autonomy into adulthood, with additional impacts on physical maturation during puberty [21].

A specific aspect of this integration is eye–foot coordination (EFC), which allows actions such as following moving objects with the eyes while performing foot and leg movements to intercept them, or maintaining balance during visually oriented tasks [19,22]. This skill enables a person to execute precise and controlled movements in response to what their eyes see, an essential ability in daily life [23]. In this context, EFC encompasses several essential cognitive processes like visual perception to interpret information, selective attention to focus on specific stimuli, and motor planning to coordinate movements [19]. Also, it involves working memory to retain and manipulate information, spatial processing to understand the location of objects, and reaction time to respond quickly [22].

Additionally, EFC also includes inhibitory control to suppress inappropriate responses and cognitive flexibility to adjust movements according to the environment [24]. Furthermore, in EFC, visual attention plays a crucial role in selecting important areas, facilitating perception, and guiding movements, as the brain prioritises visual information to coordinate subsequent motor actions [25]. Similarly, saccadic eye movements, which are rapid and simultaneous movements of both eyes in the same direction, are vital for visual search and orientation [26]. Therefore, typical EFC performance builds the basis for future tasks requiring precision, such as driving, as well as improving spatial awareness and reaction time, which are essential for daily activities and sports [23]. It also helps to identify atypical development in children [27].

In this context, EFC plays a crucial role, because if children perceive low motor competence from childhood, they are unlikely to persist, which can lead to demotivation, lack of adherence to physical activity, and sedentary lifestyles [28]. Previous research has highlighted that motor skills such as EFC are essential for athletic performance, and also for cognitive development by enhancing attention, spatial awareness, and reaction time [8,29]. In this context, according to skill acquisition theories, EFC is initially learned through verbal knowledge and becomes automated with practice [30], implying the use of cognitive resources to perform EFC with efficiency [24]. Stodden et al. [9] emphasised that failure to achieve typical motor skill development in middle childhood can limit the development of advanced skills, participation in physical activities and progress in later stages.

An important issue in EFC is how motor planning and execution are affected by interference between eye and foot movements [31]. For example, when kicking a ball, the eyes focus first on the ball, followed by the movements of the body and feet to strike it [32]. This sequence emphasises the coordination between visual attention and motor actions to ensure task accuracy and efficiency. Typically, in the double-task paradigm, the eyes move towards the target before the body, highlighting the coordination between visual attention and motor actions [32,33]. In this context, interference emphasises the difficulty experienced by the central nervous system in managing multiple motor or cognitive tasks simultaneously, which can affect the accuracy and efficiency of actions during complex conditions [34]. Thus, the focus of attention, whether external or internal, significantly influences motor performance and learning [35].

This interference arises from cognitive overload and competition for attentional resources, hindering the integration of visual and motor information necessary for effective foot movement coordination [36]. As a result, individuals may experience reduced accuracy, slower reaction times, and a higher risk of stumbling and falling in daily activities and sports [37]. In line with this, a study conducted by Buck et al. [38] explored the relationship between physical fitness and interference control in 74 children aged 7 to 12 years, using the Stroop test, FitnessGram, and intelligence quotient data. Results showed that older children, those with higher intelligence quotients, and those with better aerobic fitness showed better results on the Stroop test. Moreover, the Stroop test has been used in previous studies that showed that physical fitness improves cognition and executive functions [39,40,41].

In this sense, the dual-task paradigm, which involves performing two tasks simultaneously (e.g., cognitive and motor tasks), requires dividing attention between tasks [36], often resulting in reduced performance in one or both tasks due to limited cognitive resources, a phenomenon known as dual-task cost [42]. This dual cost is influenced by factors like task complexity, task mastery, age-related decline, and task prioritisation, highlighting the challenges of managing concurrent cognitive and motor demands [43,44,45]. In the dual-task paradigm, auditory and visual distractors provided an external focus, reducing the cognitive demands of the task [21]. However, assessing EFC in dual-task conditions in children presents challenges due to their slower cognitive and motor development, as well as their varying levels of attention and coordination skills [46]. Consequently, children and younger athletes may struggle to concentrate amidst distractions, as their cognitive systems are not yet mature enough to filter and process multiple stimuli efficiently [47]. Therefore, in children, providing instructions is crucial for facilitating the learning of motor skills [48].

To our knowledge, few studies have examined attentional focus in children, highlighting the importance of external focus for motor performance and automaticity [49,50]. Subara-Zukic et al. [51] studied unimodal (visual–auditory) sensory distractors using a dual-task paradigm to understand factors affecting motor performance and attention in children, whereas Saemi et al. [52] suggested that an external focus improves throwing accuracy and interceptive skills but benefits experienced children more than novices. Moreover, research on the dual-task paradigm in typically developing children is limited and has predominantly focused on gait-related tasks [45,53,54] or balance [55].

As regards sex differences, there is no uniform consensus on the existence of differences between boys and girls in the domain of fundamental motor skills and motor competence. Previous studies have identified sex differences [10,56] indicating that boys tend to have higher competence in object control skills than girls [10,57]. Nevertheless, other studies have shown that there are no differences between sexes, with a similar mastery of locomotor skills [58,59]. In contrast, other studies showed that girls have greater control over fundamental movement skills and motor competence [58,60]. Therefore, studies are needed to clarify these contradictory results, highlighting a gap in the understanding of fundamental motor skills by sex.

Childhood is a critical period for physical, motor, and cognitive development, during which EFC evolves significantly due to skill refinement [21]. However, assessing EFC in children, particularly within sports contexts, remains challenging due to the subjectivity of current methods, high costs, and complex testing processes. This underscores the need for reliable, portable, and standardized EFC assessments to enhance coordination evaluation, identify motor disorders, and support the recognition of talented children [61]. Therefore, this study aimed to verify the validity and reliability of the innovative EFC test in a dual-task paradigm in children aged 6–11 years and the capacity of this test to discriminate between sex and age. We hypothesised that the EFC test designed within the dual-task paradigm would demonstrate good validity and reliability for assessing prepubertal children.

## 2. Materials and Methods

### 2.1. Participants

This cross-sectional study included 440 schoolchildren aged 6 to 11 years (mean age = 8.61 ± 1.50 years). The present study involved 5 schools from rural and urban areas in southern Spain. The schools were selected by convenience sampling, according to their availability and willingness to take part in this study.

Children were eligible to participate in the present study by meeting the following inclusion criteria: (a) being enrolled in the school; (b) not having any physical (e.g., motor), sensory (e.g., visual, hearing or language), intellectual (e.g., Down syndrome), or mental disabilities; (c) having submitted informed consent signed voluntarily by the parents before starting the study. In contrast, the exclusion criteria were as follows: (a) the presence of medical conditions, injuries, or neurological or cognitive disorders affecting motor or cognitive performance; (b) the absence of attendance at assessment sessions. The initial number of participants included 461 children; however, 21 children (9 boys and 12 girls) were excluded as they did not meet the selection criteria due to medical, cognitive, and motor impairment reports provided by teachers at the school. The study received approval from the Ethics Committee of the University of Jaén (Code: JUN.21/7.TES) and adhered to the Declaration of Helsinki (2013) ethics guidelines.

### 2.2. Materials and Testing

#### 2.2.1. Anthropometric Measurements

Children were weighed with a weighing scale (Seca 899, Hamburg, Germany), and their height (cm) was measured barefoot with a stadiometer (Seca 222, Hamburg, Germany). In addition, their waist circumference (WC) was measured at the umbilical location by using a non-elastic ergonomic circumference measuring tape (Seca 201, Germany; range: 0–150 cm; accuracy: 1 mm). Body Mass Index was calculated as weight (kg)/height^2^ (m). The assessment took place during physical education classes with the assistance of the teacher. In addition, a self-assessment questionnaire (graphs) was used to assess the pubertal stages [62] according to Tanner’s [63] criteria. The results showed that all children were classified as Tanner stage 1 (prepubertal).

#### 2.2.2. Eye–Foot Coordination (EFC) Tests

The EFC test and interference modalities were designed and evaluated by a panel of four experts using the Delphi method [64,65]. The expert panel consensus achieved a concordance index of 0.759 (Fleiss’ Kappa).

**Figure 1:**
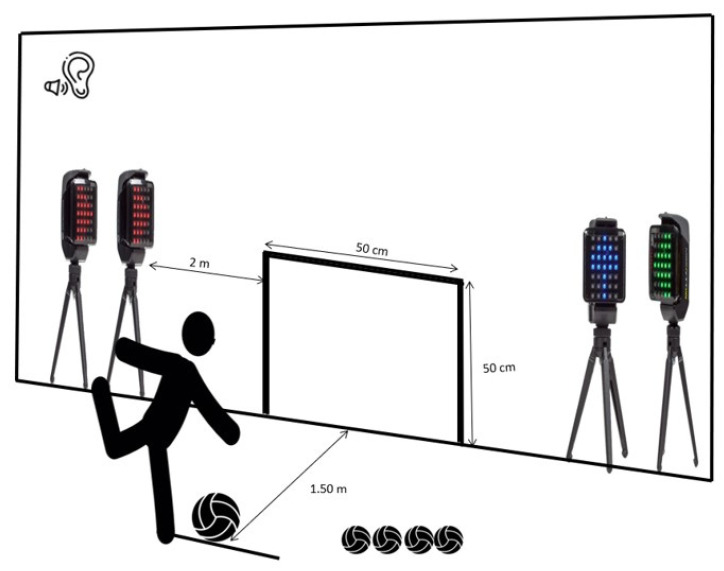
EFC test with auditory and visual interference (dual-task conditions).

EFC tests measure a child’s competence in kicking a ball and catching it back with the foot after a bounce off a wall. Children in the EFC test in this study kicked a volleyball consecutively with their foot (participants could choose the right or left foot depending on which one had greater ability) towards a marked zone on a wall as fast as possible over a period of 90 s (Figure 1).

During the assessment of the EFC test, the score was recorded based on two indicators: (1) the number of “successes” (when balls entered the marked zone) and (2) the number of “mistakes” (outside the marked zone). Several balls were available to the children to avoid wasting time if the child lost control of the ball. This test was designed by an expert panel (Cohen’s Kappa = 0.796) [64] to assess manipulative skills in children, emphasising its relevance to ball-kicking abilities and ensuring its reliability and objectivity. Although the test seems simple, it demands complex skills, including accurate foot timing, assessment of ball speed and direction, and strength.

#### 2.2.3. Auditory Interference (AI)

A verbal fluency task was used to measure participants’ speed of language production with 31 simultaneous questions of progressive difficulty (e.g., “Tell me four colours, four countries, four fruits”, etc.) [53]. Each participant answered a series of questions during the 90 s of the EFC test. Note that participants were assessed individually to avoid bias and external distractions. The questions were played through loudspeakers at 80–90 dB, located 1.5 m away from the participants. This methodology was based on previous studies that sought to assess the impact of auditory interference on attention [66].

#### 2.2.4. Visual Interference (VI)

Visual interference was assessed by displaying red, blue, and green stimuli using a Witty SEM (WITTY^®^, Microgate Srl; Bolzano, Italy) [67]. The test consisted of calling out the colour “red” each time it appeared while running the EFC test. The stimuli were programmed to appear for 1 s. In addition, a photocell was used to send the input signal to the Witty SEMs during the 90 s of the EFC test.

In addition, to determine the phenomenon of interference, closely linked to inhibitory control processes, the Stroop test was used [68]. This instrument consists of three slides with 100 items per slide. In the first slide, participants read aloud the words “RED”, “GREEN”, and “BLUE” printed in black ink. In the second slide, children name the colours in which “XXXX” was written (blue, green, or red). In the third slide, the task consists of naming the colour of the ink with which each word is printed, creating a constant interference (making the colour visible versus naming the printed colour). Each task has a time limit of 45 s. The Stroop test provides the following scores: (a) first slide, correct number of words read (“P” word); (b) second slide, correct number of colours read (“C” colour); and (c) third slide, correct number of items read (“PC” colour–word). In addition, the Stroop test had the following Cronbach’s alphas: P = 0.78, C = 0.75, and PC = 0.78 [69]. The total scores reflected the ability to control cognitive interference and inhibitory control. In addition, the scores obtained by the participants on the Stroop test variables are reported in Section 3.

### 2.3. Procedure

First, a schoolteacher informed the researchers about children who could not participate due to previous reports of physical or cognitive problems. Assessments were carried out in five different sessions at the school. In the first session, anthropometric measurements were recorded, and the Stroop test was completed. In the second to fifth sessions, the evaluation of the EFC test was started. The test was assessed in three different conditions: (1) without interference (WI), (2) with auditory interference (AI), and (3) with visual interference (VI). The children were randomised, and to avoid bias or distractions, the assessments were carried out in different spaces (without noise). The time set for each condition was 90 s, and each child made one attempt in each condition. During the assessment, support staff assisted the children with ball replacement during the test. Additionally, before the assessment, the teacher conducted a demonstration, followed by two familiarization trials per child. In the EFC test, the numbers of successes and mistakes were recorded for each condition. Finally, a retest of the EFC test was assessed after one week.

### 2.4. Statistical Analyses

This study’s data were analysed with SPSS (Version 25.0) for Windows (SPSS Inc., Chicago, IL, USA), and the level of significance was set at *p* < 0.05. Prior to starting the respective analyses, the normal distribution and homogeneity tests (Kolmogorov–Smirnov and Levene’s test, respectively) were performed.

Findings are expressed as means, standard deviations (SDs), and percentages (%). We aimed to assess the validity and reliability of the EFC test by evaluating both relative reliability (test–retest consistency) and absolute reliability, which includes the standard error of measurement (SEM) and the minimum detectable change (MDC). The SEM was calculated as the SD of the mean differences between test and retest divided by √2 [70]. The quality of the SEM was categorised as “very good”, < 5% of the total score, “good”, between 5% and 10%, “doubtful”, between 10% and 20%, and “negative”, above 20% [71]. The MDC establishes the limits within which changes in the measurement score can be attributed to measurement error. It is calculated as 1.96 × √2 × SEM [72]. To express the SEM and MDC as percentages, the following formula was used: SEM% or MDC% = (SEM or MDC/mean) × 100, where the mean is the average of the test and retest scores [73]. In addition, relative reliability was assessed using the intraclass correlation coefficient (ICC) and Pearson’s correlation (r), together with Bland–Altman plots to show agreement between the two tests (day 1 and day 2) and to detect outliers. Outliers identified were reviewed according to predefined criteria. In addition, systematic bias was considered in the interpretation of the results. A 95% confidence interval (CI) of the mean difference was used to identify systematic bias.

Differences between conditions were performed using the Mann–Whitney U test and the Kruskal–Wallis test, and post hoc analysis was performed using the Mann–Whitney U test. To analyse the relationship between various parameters, partial correlation analysis (adjusted for age and sex) was used. The magnitude of the correlation between measurement variables was designated as <0.1 (trivial), 0.1–0.3 (small), 0.3–0.5 (moderate), 0.5–0.7 (large), 0.7–0.9 (very large), and 0.9–1.0 (almost perfect) [74]. The significance level was set at *p* < 0.05.

## 3. Results

Regarding absolute reliability, the SEM for the standing successes was 3.082 (10.81%), and the MDC was 4.860 (17.05%). For the standing mistakes, the SEM was 1.551 (19.33%) and the MDC was 3.452 (43.04%). The test–retest analysis for the foot successes showed an initial mean of 28.73 ± 19.30, and the mean of the retest was 28.26 ± 19.69; *p* = 0.477. In contrast, for foot mistakes, the initial mean was 7.45 ± 2.94, while the mean of the retest was 8.59 ± 3.24, *p* < 0.001, per the difference.

**Figure 2:**
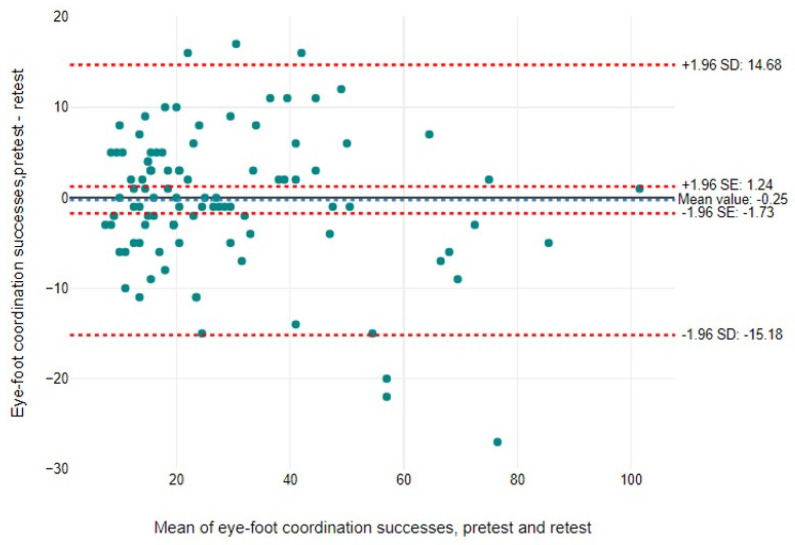
Bland–Altman graph of EFC successes: pretest/retest.

**Figure 3:**
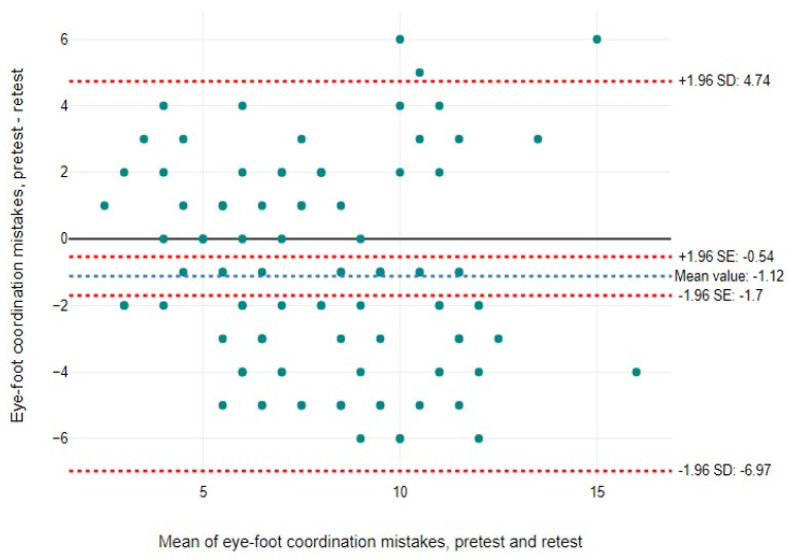
Bland–Altman graph of EFC mistakes: pretest/retest.

The ICC was 0.975 for foot successes (95% CI = 0.961, 0.983; *p* < 0.001) and 0.747 for foot mistakes (95% CI = 0.611, 0.835; *p* < 0.001). Pearson’s correlation analysis also showed a significant correlation between the initial test and retest for both foot successes (r = 0.901; *p* < 0.001) and foot mistakes (r = 0.605; *p* < 0.001). In addition, the Bland–Altman plots revealed limits of agreement (±2 SD) of 14.68 and −15.18 for foot successes, and 4.74 and −6.97 for foot mistakes (Figure 2 and Figure 3). There were six participants with foot successes and three with foot mistakes outside these limits, indicating good overall agreement between the test and retest sessions.

The socio-demographic data, the Stroop test, and the EFC test in the three conditions (WI, AI, VI) by sex are displayed in Table 1. Boys had a significantly higher number of foot successes in the WI condition (*p* < 0.001), AI condition (*p* < 0.001), and VI condition (*p* < 0.001). In addition, girls made significantly more foot mistakes in the VI condition compared to boys (*p* = 0.025). Also, boys showed a higher percentage of dual-task cost success during the VI condition compared to girls (*p* = 0.016). These results indicate significant differences in EFC performance between boys and girls.

**Figure 4:**
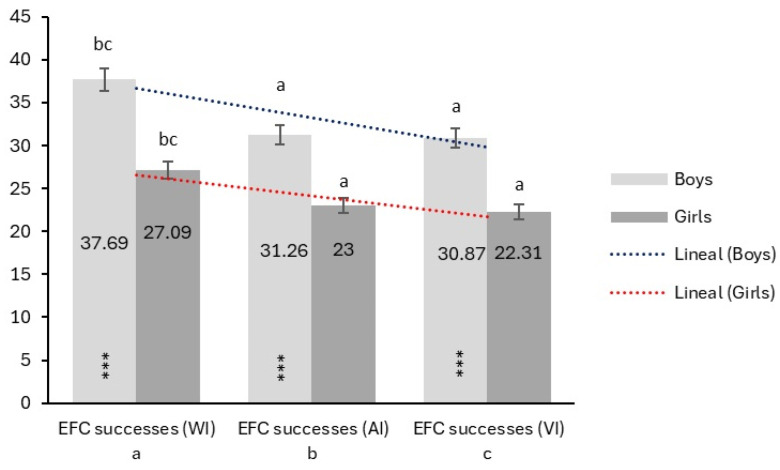
Linear trends in the number of successes on the EFC test between sexes in each condition. WI = without interference; AI = auditory interference; VI = visual interference; *** Denote differences between sexes (*p* < 0.001); Lowercase letters denote significant differences between conditions.

Figure 4 displays the differences between boys and girls in the numbers of successes on the EFC test in the WI, AI, and VI conditions. Boys had a significantly higher number of successes in the WI (*p* < 0.001), AI (*p* < 0.001), and VI (*p* < 0.001) conditions than girls. The dotted lines show the linear trends for the boys (blue line) and girls (red line). The linear trends of successes decreased from WI to VI for both sexes. The slope of decline was less for boys than for girls. In addition, boys maintained more successes than girls across all conditions, showing greater consistency in performance.

**Figure 5:**
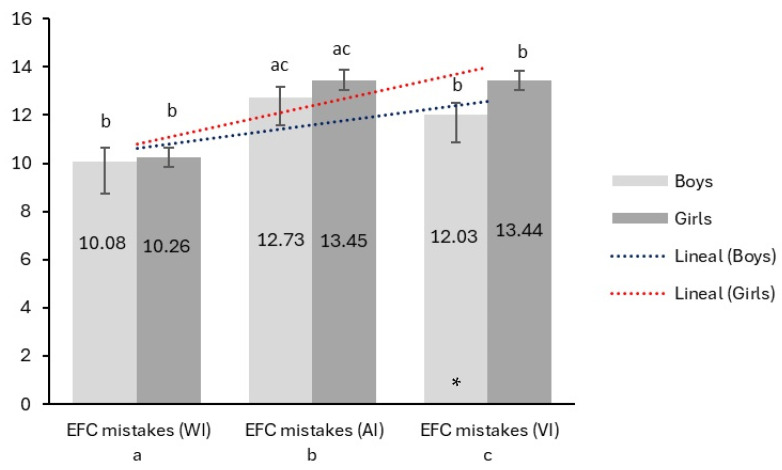
Linear trends in the number of mistakes on the EFC test between sexes in each condition. WI = without interference; AI = auditory interference; VI = visual interference; * Denote significant differences between sexes (*p* < 0.05); Lowercase letters denote significant differences between conditions.

The mistakes in the three conditions (WI, AI, VI) of the EFC test between boys and girls are shown in Figure 5. The results indicate that, in the VI condition, girls made significantly more mistakes than boys (*p* = 0.025). The linear trends of mistakes increased from WI to VI for both sexes. The slope of incline was greater for girls than for boys. Girls made more mistakes than boys across all conditions, indicating greater variability in their performance.

The partial correlation analysis (sex-adjusted) revealed significant associations between age and successes in the WI (r = 0.441, *p* < 0.001), AI (r = 0.413, *p* < 0.001), and VI (r = 0.400, *p* < 0.001) conditions, as well as regarding mistakes in the AI condition (r = 0.259, *p* < 0.001). Moreover, several correlations were found between EFC performance and the Stroop test. For example, EFC successes in the WI condition were associated with word score (r = 0.312, *p* < 0.001) and words/colours score (r = 0.295, *p* < 0.001). Likewise, EFC successes in the VI condition showed an association with word score (r = 0.386, *p* < 0.001) and words/colours score (r = 0.284, *p* < 0.001). Regarding daily time spent in extracurricular physical activities, a bivariate correlation analysis revealed a significant association with performance in the WI (r = 0.183, *p* < 0.05), AI (r = 0.195, *p* < 0.05), and WI (r = 0.210, *p* < 0.05) conditions.

**Figure 6:**
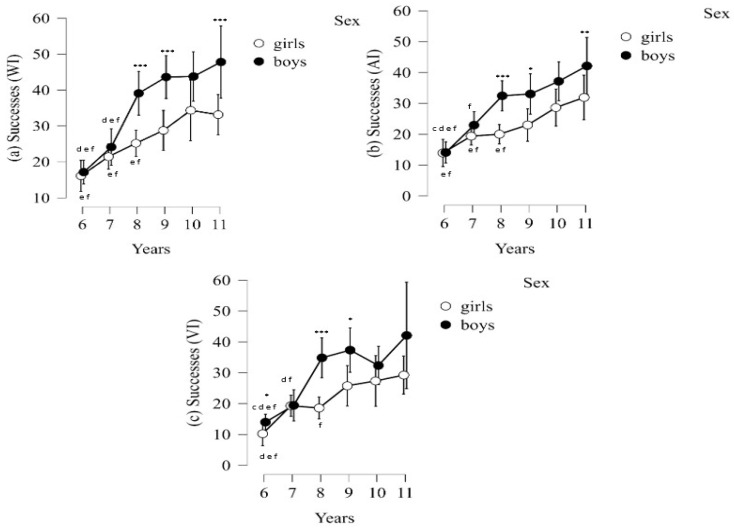
Interaction of sex and age in EFC successes in the three conditions (WI, AI, VI). WI = without interference; AI = auditory interference; VI = visual interference; * Denote significant differences between sexes (* *p* < 0.05, ** *p* < 0.01, *** *p* < 0.001). Lowercase letters denote significant differences between ages of each sex.

Information regarding the interaction of sex and age on EFC test success performance within the three conditions (WI, AI, VI) is presented in Figure 6. The results showed that boys consistently outperformed girls in EFC successes in all three conditions (*p* < 0.001, respectively). For example, boys aged 8, 9, and 11 years showed a higher number of successes in the WI condition (*p* < 0.001, respectively) and in the AI condition (*p*-values ranged from <0.001 to 0.020) than girls. Likewise, in the VI condition at ages 6, 8, and 9 years (*p*-values ranged from <0.001 to 0.022), boys were significantly superior.

In contrast, significant differences in mistakes were found in the VI condition (*p* = 0.017), with girls showing more mistakes than boys. In terms of age, both sexes show a general trend of increasing EFC successes with age (*p* < 0.001). Significant differences between the ages of each sex are indicated by lowercase letters in Figure 6.

## 4. Discussion

The main findings of the current study indicate that the EFC test demonstrated adequate test–retest reliability within a dual-task condition incorporating auditory and visual distractors, making it a reliable tool for assessing EFC performance in prepubertal children aged 6 to 11 years. Furthermore, the test effectively distinguishes between sex and age groups, which supports its validity for a variety of applications.

Regarding age, EFC performance increased due to natural maturation processes. In terms of dual-task cost, sex differences were only observed in the cost of the VI condition, with boys showing higher values than girls. However, the dual-task cost was not influenced by age. Finally, a relationship was identified between EFC performance and inhibitory control processes, as well as between daily extracurricular physical activity time and performance in each of the EFC test conditions.

Concerning our results on sex differences, boys exhibited better performance across all conditions of the EFC test compared to girls. Similarly, a systematic review conducted by Bolger et al. [10] showed that fundamental skills and motor competence increase with age throughout childhood and that boys, from childhood onwards, demonstrate higher levels of control in object control skills than girls. One possible explanation is that children who participate in sporting activities demonstrate better motor skills, which highlights the importance of the role of schools in their development and the need to plan effective strategies to improve teaching in physical education [13]. In addition, our findings seem consistent with Barnett et al. [57], who found boys to be more proficient in object control versus girls, who instead excelled in balance and fine coordination; furthermore, competence in object control in childhood significantly predicts competence in adolescence. Similarly, Piek et al. [50] found that children who participated in activities involving EFC, such as jumping and playing ball games, showed significant improvements in gross motor skills. This result highlights the importance of focusing on coordination exercises to condition and enhance motor skills in students [75,76]. Offering a variety of motor activities helps to ensure the acquisition of a wide range of motor skills and fosters the development of coordination [77]. In contrast to our results, Butterfield et al. [78] found no significant differences between sexes, despite boys outperforming girls in throwing and kicking.

Regarding the interaction between sex and age, boys consistently had more successes than girls across all conditions assessed (WI, AI, VI), with significant differences at multiple ages. Both sexes showed an increase in successes with age, although girls showed greater variability and a more gradual increase. These differences suggest a significant interaction between sex and age in EFC test performance. In this line, a meta-analysis by Utesch et al. [6] concluded that there is a moderately positive relationship between motor competence and physical fitness, which becomes stronger and increases with age.

In addition, the correlational analyses indicated that age was significantly associated with EFC successes across all conditions (WI, AI, VI) and with mistakes in the AI condition, regardless of sex. Furthermore, EFC successes in all three conditions correlated with Stroop test scores. These findings suggest an interrelationship between age, EFC performance, and the ability to manage attention, cognitive flexibility, and cognitive interference as measured by the Stroop test. In this context, EFC skill requires concentration and attention, skills that improve school performance and promote sensory integration, especially benefiting children with learning difficulties or developmental disorders [79,80].

In this context, a systematic review by van der Fels et al. [81] explored how motor and cognitive skills are interrelated in children aged 4–16 years, concluding that motor skills, especially fine motor skills and bilateral coordination, are significantly associated with cognitive performance. Similarly, Chang et al. [82] concluded that their coordination exercises improved the allocation of attentional resources and the efficiency of neurocognitive processing in children after 16 weeks of intervention using coordination exercises of different intensities. The importance of neuromuscular coordination in different human movements lies in the learning and development of skills for the autonomous development of daily activities [24]. Likewise, a meta-analysis assessed the relationship between motor competence and executive functions in children and adolescents, showing that greater competence was associated with better performance in executive functions including inhibition, working memory, and cognitive flexibility [7]. This study underlines the importance of promoting motor competence from an early age to improve cognitive functions in child development. In addition, the positive association between extracurricular physical activity time per day and performance in the conditions of the EFC test suggests that participation in physical activities might be linked to better performance in dual-task conditions and improvement in motor skills. These results match those observed in earlier studies [13,83]. Therefore, motor skills, especially fine motor skills and coordination, are crucial for cognitive development in children. Promoting these skills through targeted activities can significantly enhance cognitive performance, highlighting the need to integrate motor skill development in early childhood programmes [84]. Overall, our findings have important implications for the design of educational and sports programmes that incorporate EFC activities, benefitting both typically developing children and those with learning difficulties. Promoting EFC through games, specific exercises, and training technologies can significantly improve children’s overall development [10].

### 4.1. Study Strengths and Limitations

This study had several limitations, including that its cross-sectional design did not allow us to establish cause–effect relationships between age, cognitive function, and dual-task cost over the course of child development. In addition, there were no inclusions of critical variables such as the children’s level of PA, their physical fitness, previous sports skills of the children, or the influence of external factors. Conversely, although participants were instructed not to focus on the EFC test or the dual-task conditions, the lack of clarity about how they prioritised the tasks may have influenced our results.

However, a notable strength of this study is its ecological validity, as the assessments were conducted in a school setting, which increases the relevance of the findings to real-world settings. We grant, however, that the field could benefit from larger and more diverse samples, longitudinal studies, and an interdisciplinary approach to address the complexities of EFC and maximise its practical applications in education and sports.

### 4.2. Practical Applications

To our knowledge, there were no standardised protocols to assess EFC in dual-task conditions, so we proposed an ecological protocol for school and sports contexts, integrating routine motor activities and assessment indicators like accuracy, reaction time, and general coordination in children. This approach is the first to analyse the influence of age and sex in a school population, seeking to improve motor and cognitive development. The approach might, therefore, improve the accuracy and applicability of assessments of early childhood coordination.

## 5. Conclusions

The results of the current study showed that the EFC test had adequate test–retest reliability. Likewise, the test proved to be reliable in discriminating sex and age differences in prepubertal children. In addition, children’s performance in the dual-task condition tended to worsen with the introduction of interference (auditory and visual), regardless of sex and age. Furthermore, considering that physical education classes play a key role in improving EFC and motor competence, this ecologically designed test is positioned as an effective tool for assessing early motor development. In addition, EFC tests can be used to identify children with atypical development as well as those with exceptional potential. Therefore, teachers, coaches, and other staff working with children of this age group might use this test even with limited materials and technological resources.

## Figures and Tables

**Table 1:** Anthropometric measures, Stroop test, and EFC test within three conditions according to sex.

Variables	All N = 440 Mean (SD)	Boys N = 223 Mean (SD)	Girls N = 217 Mean (SD)	*p*-Value
Age (years)	8.61 (1.50)	8.56 (1.48)	8.65 (1.52)	0.638
Body weight (kg)	33.55 (10.41)	34.04 (10.75)	33.06 (10.07)	0.366
Height (m)	135.05 (11.54)	135.30 (11.85)	134.79 (11.24)	0.603
Body Mass Index (kg/m^2^)	18.08 (3.75)	18.25 (3.65)	17.92 (3.86)	0.145
Waist circumference (cm)	63.98 (10.28)	64.61 (10.19)	63.35 (10.36)	0.128
Extracurricular physical activity time per day (hours)	1.37 (1.05)	1.50 (1.13)	1.16 (0.91)	0.231
Stroop test				
Words (n)	87.41 (38.70)	87.45 (47.37)	87.38 (27.32)	0.290
Colours (n)	72.89 (53.55)	73.25 (71.01)	72.53 (25.87)	0.093
Words–colours (n)	40.88 (19.54)	40.56 (20.04)	41.21 (19.05)	0.754
Interference (s)	−1.49 (24.71)	−0.14 (22.27)	−2.86 (26.97)	0.144
Eye–foot coordination test				
# Foot successes (WI)	32.37 (18.33)	37.69 (19.89)	27.09 (14.91)	<0.001
# Foot mistakes (WI)	10.17 (7.16)	10.08 (8.36)	10.26 (5.75)	0.101
# Foot successes (AI)	27.20 (16.10)	31.26 (17.48)	23.00 (13.35)	<0.001
# Foot mistakes (AI)	13.08 (6.46)	12.73 (6.75)	13.45 (6.13)	0.163
# Foot successes (VI)	26.84 (15.98)	30.87 (17.54)	22.31 (12.65)	<0.001
# Foot mistakes (VI)	12.69 (6.60)	12.03 (6.98)	13.44 (6.10)	0.025
% Dual-task cost—successes (AI)	−9.33 (38.67)	−10.85 (39.86)	−7.75 (37.45)	0.487
% Dual-task cost—mistake (AI)	65.79 (135.01)	76.29 (167.16)	54.88 (89.60)	0.751
% Dual-task cost—successes (VI)	13.55 (110.61)	19.26 (94.34)	7.14 (126.57)	0.016
% Dual-task cost—mistake (VI)	54.32 (134.84)	68.01 (160.26)	38.83 (96.95)	0.461

WI = without interference; AI = auditory interference; VI = visual interference; # = number of successes or mistakes in each condition; % = percentage.

## Data Availability

The data that support the findings of this study are available from the corresponding author, upon reasonable request.
